# The effect of screen time on the presentation and treatment of primary monosymptomatic nocturnal enuresis

**DOI:** 10.1186/s12894-023-01184-y

**Published:** 2023-02-17

**Authors:** Arif Demirbas, Hacer Gizem Gercek

**Affiliations:** 1 Department of Urology, Afyonkarahisar Health Sciences University, Dörtyol, 2070. Sokak NO: 3/4, F Block, 03030 Afyonkarahisar Merkez/Afyonkarahisar, Turkey; 2 Department of Child and Adolescent Psychiatry, Afyonkarahisar Health Sciences University, Dörtyol, 2070. Sokak NO: 3/4, A Block, 03030 Afyonkarahisar Merkez/Afyonkarahisar, Turkey

**Keywords:** Nocturnal enuresis, Screen time exposure, Treatment of enuresis

## Abstract

**Background:**

We aimed to investigate if there was any relationship between screen time (ST) and the severity of primary monosymptomatic nocturnal enuresis (PMNE) and treatment success.

**Methods:**

This study was conducted in urology and child and adolescent phsychiatry clinic in Afyonkarahisar Health Sciences University Hospital. After diagnosis patients were seperated by the ST for exploring causation. Group 1 > 120, Group 2 < 120 (min/day). For the the treatment response, patients were grouped again. Group 3 patients were administered 120 mcg Desmopressin Melt (DeM) and were requested < 60 min ST. Patients in Group 4 were given 120 mcg DeM solely.

**Results:**

The first stage of the study included 71 patients. The ages of the patients ranged from 6 to 13. Group 1 comprised 47 patients, 26 males and 21 females. Group 2 comprised 24 patients,11 males and 13 famales. Median age was 7 years in both groups. The groups were similar in respect of age and gender (*p* = 0.670, *p* = 0.449, respectively). A significant relationship was determined between ST and PMNE severity. Severe symptoms were seen at the rate of 42.6% in the Group 1, and at 16.7% in the Group 2 (*p* = 0.033). 44 patients completed the second stage of the study. Group 3 comprised 21 patients, 11 males and 10 females. Group 4 comprised 23 patients,11 males and 12 famales. Median age was 7 years in both groups. The groups were similar in respect of age and gender (*p* = 0.708, *p* = 0.765, respectively). Response to treatment was determined as full response in 70% (14/20) in Group 3 and in 31% (5/16) in Group 4 (*p* = 0.021). Failure was determined in 5% (1/21) in Group 3 and in 30% (7/23) in Group 4 (*p* = 0.048). Recurrence was determined at a lower rate in Group 3 where ST was restricted (7% vs. 60%, *p* = 0.037).

**Conclusion:**

High screen exposure may be a factor for PMNE aetiology. And also reducing ST to a normal range can be an easy and beneficial method for treatment of PMNE.

*Trial Registration* ISRCTN15760867(www.isrctn.com). Date of registration: 23/05/2022. This trial was registered retrospectively.

## Background

Monosymptomatic nocturnal enuresis, commonly known as bedwetting, is defined as intermittent night-time bedwetting only, without any daytime lower urinary tract symptoms. When this continues without a 6-month dry period, it is known as primary monosymptomatic nocturnal enuresis (PMNE). This is accepted physiological up to the age of 5 years and for cases older than 6 years, treatment is recommended [1, 2].

Three important pathophysiological factors are responsible for PMNE. Two of these factors are an increase in respect of night-time in the day-night urine output ratio, and reduced nocturnal functional bladder capacity. However, the main pathophysiology is believed to be an increased threshold of waking from sleep despite the fullness of the bladder. There has been seen to be a difference in REM sleep in bedwetting children compared to normal children, which has been proven in sleep disorder laboratory tests. Therefore, many reasons which could lead to sleep disorders, primarily adenotonsillar hypertrophy have been the subject of research for the etiology of PMNE [3–6]. In recent studies, screen time (ST) exposure has been reported to be a factor that could lead to sleep disorders. It has been determined that excessive screen time (EST) which could lead to emotional, cogntive and behavioural problems, also impairs sleep quality [7, 8].

With the consideration that screen time could be a pathophysiological factor of sleep disorder in children diagnosed with PMNE, the aim of this study was to investigate whether there was any relationship between screen time and the degree and severity of PMNE and treatment success.

## Methods

**Fig. 1:**
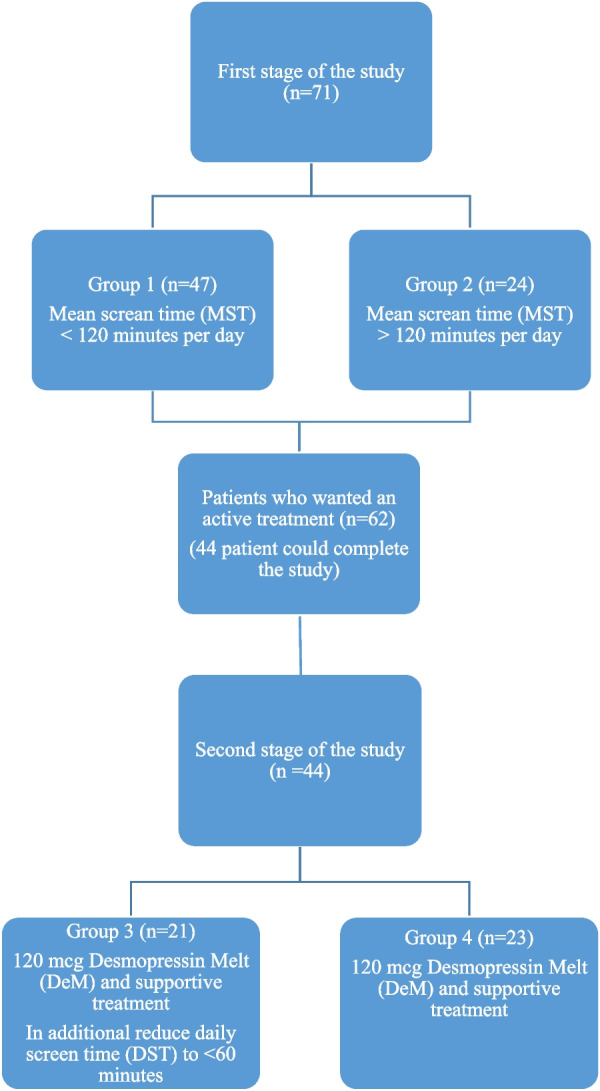
Flow chart for first stage and second stage study

This study was conducted in urology and child and adolescent phsychiatry clinic in Afyonkarahisar Health Sciences University Hospital. After ethical approval (Afyonkarahisar Health Sciences University Clinical Research Ethics Committee. 2011-KAEK-2, 2021/198) we recorded the data prospectively (Clinical trial registration: ISRCTN15760867. 23/05/2022). Written informed consent was obtained from the parents or legal guardian of the participation include the study.. The study included the families of patients who presented because of bedwetting and were diagnosed with PMNE from the history, a 2-day complete voiding-drinking diary and urine analysis. All the families participated voluntarily and no patient had previously received treatment. Patients were excluded if they had any neurological disease, obstructive respiratory tract disease, diabetes mellitus, or body mass index > 95th percentile and reduced bladder capacity detected by voiding diary (Koff Formula; Bladder capacity (ounces) = age (years) + 2) [9]. After the diagnosis of PMNE, the parents were requested to keep a diary for 1 week of the minutes of screen time (ST) (television, tablet, computer, mobile telephone, video game console). At the first stage of the study, feedback was obtained from 71 families. To determine the relationship between ST and the degree and severity of PMNE symptoms, the patients were separated into two groups as Group 1 with mean screen time (MST) > 120 min per day and Group 2 with < 120 min MST per day (Fig. 1).

The age and gender of each patient was recorded and PMNE was classified as described in previous studies as mild (1–2 wet nights/week), moderate (3–5 wet nights/week) and severe (6–7 wet nights/week).^4^ The mean number of wet nights per week were recorded and the groups were compared according to these parameters.

Patients who wanted an active treatment who were not willing to take the conservative “wait and see” approach were also included in the study to see the effect of ST restriction in the treatment of PMNE. Thus, at the second stage, 62 of 71 patients were included, but only 44 could complete the study. Patients were allocated to 2 groups using randomly generated numbers with a computer software program (www.Randomizer.org). Group 3 patients were administered 120 mcg Desmopressin Melt (DeM) and in addition to supportive treatment were requested to reduce daily screen time (DST) to < 60 min. Patients in Group 4 were given 120 mcg Desmopressin Melt (DeM) and supportive treatment was recommended (Fig. 1).

The response to treatment was measured at the end of 3 months. Full response was defined as 100% dryness, partial response as 50–99% dryness and failure as < 50% dryness. For the patients with full response in both groups, the DeM treatment was terminated and at a follow-up visit 1 month later they were questioned about recurrence. The groups were statistically compared in respect of descriptive data, response to treatment and recurrence.

### Statistical analyses

The statistical analyses were performed using the Statistical Package for the Social Sciences (SPSS.21.0) software. The distribution of pretreatment descriptive data and the posttreatment values was determined with the Kolmogorov–Smirnov test. For independent variables with parametric distribution, the t-test (mean ± std) was applied and for variables with non-parametric distribution, the Mann Whitney U-test (median, min–max). Chi-square and Fisher’s exact tests were used for qualitative data. A value of *p* < 0.05 was regarded as statistically significant.

## Results

The first stage of the study included 71 patients ranging in age from 6 to 13 years. Group 1 comprised 47 patients with MST 142.27 ± 23.32 min and Group 2 comprised 24 patients with MST 90.25 ± 15.68 min. The groups were similar in respect of age and gender (*p* = 0.670, *p* = 0.449, respectively). In the comparison of symptom severity, in Group 1, 19.1% of patients were determined with mild, 38.3% with moderate and 42.6% with severe PMNE. In Group 2, 33.3% were determined with mild, 50.0% with moderate and 16.7% with severe PMNE. The difference between the groups was statistically significant (*p* = 0.033). The mean number of wet nights was 4.61 ± 1.73 in Group 1 and 3.79 ± 1.74 in Group 2, with no statistically significant difference between the groups (Table 1).

**Table 1:** Comparison of groups for age, gender, severity and frequency of PMNE symptoms

	GROUP 1 (n = 47)	GROUP 2 (n = 24)	*p*
Age (median, min–max)	7 (6–12)	7 (6–13)	0.670
Gender (M/F)	26/21	11/13	0.449
Screen time (min/day)	142.27 ± 23.32	90.25 ± 15.68	< 0.001
Severity of symptoms			
Mild	9 (19.1%)	8 (33.3%)	
Modarate	18 (38.3%)	12 (50%)	0.033
Severe	20 (42.6%)	4 (16.7%)	
MNWN*	4.61 ± 1.73	3.79 ± 1.74	0.057

*MNWN* mean number of wet nights per week

In the second stage of the research, Group 3 and Group 4 were found to be statistically similar in respect of age before treatment, gender, symptom severity, number of wet nights per week and daily screen time (DST) (*p* = 0.708, *p* = 0.765, *p* = 0.559, *p* = 0.569, *p* = 0.532, respectively) (Table 2). After 3 months of treatment and restricted screen time, median ST was calculated as 52 min (range, 30–60 min) in Group 3 and 120 min (range, 76–180 min) in Group 4 (*p* < 0.001).

**Table 2:** Descriptive data, response to treatment, and recurrence rates of the groups

	GROUP 3 (n = 21)	GROUP 4 (n = 23)	*p*
Age (years) (median, min–max)	7 (6–12)	7 (6–13)	0.708
Gender (M/F)	11/10	11/12	0.765
Symptom severity (moderate/severe)	11/10	13/10	0.559
Pre-treatment number of wet nights (median, min–max)	6 (3–7)	5 (3–7)	0.569
Pre-treatment daily screen time (median, min–max)—mins	135 (90–210)	126 (68–185)	0.532
Post-treatment daily screen time (median, min–max)—mins	52 (30–60)	120 (76–180)	< 0.001
Full response to treatment %(n)	70% (14/20)	31% (5/16)	0.021
Failure of treatment %(n)	5% (1/21)	30% (7/23)	0.048
Recurrence %(n)	7% (1/14)	60% (3/5)	0.037

Patients in Group 3 reported complying with the rule of DST < 60 min throughout the study. Response to treatment measured at 3 months was determined as full response in 70% (14/20) in Group 3 and in 31% (5/16) in Group 4 (*p* = 0.021). Failure in response to treatment was determined in 5% (1/21) in Group 3 and in 30% (7/23) in Group 4 (*p* = 0.048) (Table 2). Recurrence at 1 month after the completion of treatment was determined at a lower rate in Group 3 where DST was restricted (7% vs. 60%, *p* = 0.037) (Table 2).

## Discussion

According to the American Academy of Pediatrics guideline, ST exposure for children aged 2–5 years should be limited to 1 h per day. This limitation is relaxed for children over 5 years of age taking daily physical activity (1 h) and sleep duration (8–12 h) into consideration and under family controlled conditions of content and not sleeping in the same room as media devices (telephone, tablet, television etc.) [10]. With developing technology and digital devices, the ST exposure of children has beome an increasing problem throughout the world [11, 12]. In addition to being a family problem, as ST can disrupt the parent–child relationship, ST has also been held responsible for many pathologies from neurodevelopmental problems of the child to sleep disorders [7, 8].

Several studies have shown that sleep disorders and an impaired waking threshold could be risk factors for PMNE [3, 13, 14]. In treatment, it is attempted to eliminate these changeable risk factors. Invasive methods such as rapid maxillary expansion and adenotonsillectomy for sleep disorders associated with respiratory tract obstruction have been reported to be successful in enuresis treatment [15, 16]. In a study by Kaya et al. [4], it was reported that even passive smoking exposure, which can be simply changed, reduces the success of DeM treatment in PMNE.

In the light of previous studies, we want to investigate the relationship between ST and bedwetting, which is known to have a psychological effect on school-age children. In the first stage of the study, a significant relationship was determined between ST and PMNE severity. Severe symptoms were seen at the rate of 42.6% in the group with more screen exposure, and at 16.7% in the group with less ST. It was then aimed to evaluate the effect of limiting ST on the success of DeM medical treatment in children who started active treatment at the request of their parents.

In important publications in literature, a full response to DeM treatment has been reported of approximately 30%, and in the current study, this rate was 31%, as expected, in patients receiving DeM and supportive treatment [17]. However, it is noteworthy that the response to treatment was 70% in the group which reduced ST from an initial median 135–52 min as a result of our recommendations in addition to the DeM and classic treatment. Similar success was obtained in the recurrence rates at 1 month after completing treatment in the group which restricted ST (7% vs. 60%). However, it is not clear whether these data determined in this study have an organic cause such as neurological sleep disorder known to be caused by excessive screen time (EST), or if this pathology is the result of the psychological motivation source of family participation in treatment, which is known to be important, as stated in previous studies [18].

There are some limitation factors in our study. For example, family history of enuresis and sociocultural status were ignored, which might affect the first part of the study. The small cohort, hight drop out rate are the other limitations.

## Conclusıons

In conclusion, bringing ST to a normal range, which should be controlled in childhood, can be considered to contribute to the treatment of PMNE, just as it will contribute to a sufficient level of physical activity in children. This method which can be easily applied and is beneficial shows a positive effect on response to treatment and recurrence. Nevertheless, there is a need for further randomised, controlled studies and meta-analyses, as required by evidence-based medical regulations, to support these results.

## Data Availability

The datasets used and/or analyzed during the current study are available from the corresponding author on reasonable request.

## Abbreviations

ST: Screen time
PMNE: Primary monosymptomatic nocturnal enuresis
EST: Excessive screen time
MST: Mean screen time
DeM: Desmopressin Melt
DST: Daily screen time
MNWN: Mean number of wet nights per week
